# Homœopathy, Allopathy, and “Young Physic”

**Published:** 1846-06

**Authors:** 


### REVIEWS.

---

ART. V.—*Homœopathy, Allopathy, and 'Young Physic.'* By JOHN FORBES, M.D., F.R.S., &c., &c. Philadelphia: Lindsay & Blakiston. 1846. 16mo. pp. 121.

About the time that Galvani made the signal observation which has perpetuated his name in connexion with a new and wonderful science, Hahnemann was engaged in translating Cullen's Materia Medica into German; and while about that task the fundamental doctrine of Homœopathy entered his imagination. Up to that period, the forty-fifth year of his life, he seems to have been reconciled to the prevalent system of the times; but the attempt of the Edinburgh professor to explain the action of bark in curing intermittent fevers appearing to him unsatisfactory, he resolved to experiment with it upon his own person. He was in the most perfect health when he entered upon these experiments; but soon he felt a change; he had scarcely taken a full dose of the medicine before he was seized with all the symptoms of ague. And "forthwith," says his historian, "arose in his mind a conception of the great truth which was destined to constitute the basis of the new art of medicine." It was a natural question, that which proposed itself;—"May not the power of cinchona to cure ague, depend on its power to excite in the healthy body a similar disease?" With the view of answering it, he made numerous trials with various medicines upon himself and others, and his report was, that they never failed to "excite in the healthy body the same symptoms which they were capable of removing where these occurred naturally in the diseased body." His next step was to look into the records of medicine, as to the effects accidentally produced by poisons and other strong drugs. Here he thought he found everything confirmatory of his peculiar views derived from experiment, and the conclusion was no longer to be resisted —"that every disease is best cured by that medicine which is capable of producing in the healthy body similar symptoms, or a like disease." Hence the doctrine of *homœopthy*—homion pathos—stated briefly, "*similia similibus curantur—Like are cured by like*, i. e., *homœopathically*."

This doctrine, we have seen, is as old as galvanism, and comes down to us from a former century. It is no upstart doctrine, that, having amused the minds of men for a few brief years, has sunk down into "a dark and long outliving night." It has survived its author, and seen a generation of men pass away from the earth; and still it exhibits no indications of decay. It has spread over the civilized world, and taken the deepest root where men esteem themselves most advanced in civilization. Everywhere instances are known of physicians who have quit the regular walks of the profession to enlist as its disciples, and their apostacy has been rewarded by a full share of public confidence. Hospitals have been erected in which to make fair and ample trial of its efficiency; kings and princes in other lands have been its patrons, and the first of American poets is a convert to its truth. Finally: it has found advocates among men of real science; physicians of learning and experience have been won over to it; and it is now entertained and taught by a professor in one of the oldest and most noted of British Universities. Our allusion is to an institution no less renowned than the University of Edinburgh, the Professor of Medicine and General Pathology in which, W. Henderson, M.D., has issued an octavo volume in defence of the system. Great as were the previous triumphs of homœopathy, this is the one which will probably be regarded by the friends of that doctrine as the most substantial, and will excite medical men to the most profound reflection.

Such a doctrine, it will be conceded, is worthy of examination—the secret, at least, of its perpetuity and success; and we are glad that it has been made, in a spirit of apparent candor and fairness, and certainly with singular ability, by Dr. Forbes, in the little volume which stands at the head of this article. Homœopathy has been occasionally adverted to, but never formally noticed in the pages of this Journal, and we therefore propose now to lay before our readers some of the conclusions respecting it, at which one of the editors of the Cyclopædia of Practical Medicine, and one of the most eminent of English physicians, has arrived, together with some of our own thoughts on the subject. We will premise that Dr. Forbes has no faith in homœpathy; still his aim, in the work before us, which appeared first in the Review of which he is editor, is not to expose, but to *examine* its pretensions.

Homœopathy stands apart from all other systems of medicine equally in doctrine and practice. All proceed alike upon experience; but while *Allopathy*, in all its branches, is founded upon the action of medicines on the sick, homœopathy has been deduced from the effects of these agents on people in health. This is one feature of homœopathy, but the most distinguishing, and by far the most marvellous one, is the infinitely small doses in which its remedies are administered. Originally, it is said, this was not a part of Hahnemann's system; but was engrafted upon it after its author found that medicines, in appreciable doses, were too powerful in their action upon the system when its susceptibilities were exalted by disease. It has been hinted that his reason for adopting it was one not so creditable to his system—that learning by frequent trials that his homœopathic remedies did injury to his patients, he found it necessary to reduce the dose until they ceased to be felt, and so became harmless. Certainly, as observed by Dr. Forbes, "there seems to be a contradiction in the facts, as well as the reasoning of Hahnemann, in regard to this matter." It is from the augmented sensitiveness of the affected part induced by disease, he says, that the remedy operates in infinitesimal doses. If this is the case, how are we to account for the multitude of symptoms —his "medicinal diseases"—recorded in his works as the results of medicines acting on the healthy body? In health this sensitiveness is not present, and yet, if we are to credit his observations on himself and others who experimented with his remedies, it was not uncommon to have a score or two of medicinal diseases flowing from one of his doses. These doses have been more ridiculed than any other part of his system, and still we verily believe that it is impossible to rise to the height of their absurdity. We quote from Dr. Forbes on this subject:

"So minute are the doses prescribed by the Hahnemannic school, that they are scarcely conceivable by the human mind. They defy all the powers of chemistry and physics to detect in them any trace of the remedial substances which they profess to contain, and they almost confound arithmetic in reckoning their amount. We are not ashamed to confess, that our own powers are inadequate to put down in figures an ordinary homœopathic dose, and we suspect that many of the homœopathists themselves would find themselves in the same predicament on trial. The following are the different *attenuations* or doses used:

| | | |
|---|---|---|
| First | =one hundredth of a drop or grain. | |
| Second | =one ten-thousandth | do. |
| Third | =one millionth | do. |
| Sixth | =one billionth | do. |
| Ninth | =one trillionth | do. |
| Twelfth | =one quadrillionth | do. |
| Fifteenth | =one quintillionth | do. |
| Eighteenth | =one sextillionth | do. |
| Twenty-first | =one septillionth | do. |
| Twenty-fourth | =one octillionth | do. |
| Twenty-seventh | =one nonillionth | do. |
| Thirtieth | =one decillionth | do. |

"The primary dilutions or attenuations are used comparatively rarely; the higher ones, as the sixth, twelfth, twentyfirst, and thirtieth, very commonly. It may be worth a moment's trouble to try how far we really understand or comprehend these numbers. Looking at the first of these we have no difficulty. The *hundredth* (100th) part of a grain, is intelligible enough; the *ten-thousandth* (10,000th) is comprehensible, but begins to waver before the mental view; while the *millionth* (1,000,000th) part of a grain, puts our powers of comprehension on the rack, and leaves us in a chaos of undefined entities or non-entities, we know not which. We fancy that we grasp the reality, and then it instantly vanishes as a phantom, even beyond the sphere of imagination itself. Having got so far, the additional subdivisions, or attenuations, scarcely add to our difficulties. The mind, in any such case, is occupied by a word more than a thing—and whether the word be a millionth, billionth, or a decillionth, the power of comprehension seems the same.—And yet the *actual difference* between these quantities is immense,—so immense as to be almost as inconceivable as the actual things themselves. This will be more intelligible, we think, by setting it down in words thus:—

| | |
|---|---|
| One thousand thousands, is - - | A Million. |
| One million millions - - - - | A Billion. |
| One million billions - - - - | A Trillion. |
| One million trillions - - - - | A Quadrillion. |
| And so on to - - - - - - | A Decillion. |

"Now, we believe this last denomination (according to the English mode of enumeration) would stand thus in figures:—

1,000000,000000,000000,000000,000000,000000,000000,000000,000000,000000,000000,000000.

"Imagine, if you can, a grain of silica, or charcoal, or oyster-shell, (powerful remedies according to Hahnemann and his followers, in this attenuated condition,) divided into this number of parts: and one of these parts is not only fit and proper dose to be given as a remedy for severe diseases, but is an agent of such potent influence on the animal economy, that *one dose* of this amount will continue acting for thirty, forty, or fifty days, and must not be interfered with by any repetition of it, for fear of deranging or destroying its curative virtue! Thus, Hahnemann tells us that a sextillionth of a grain of carbonate of ammonia will act beneficially upwards of thirty-six days; that the decillionth of a grain of oyster-shell (*calcarea*) will require forty, fifty, and even more days, 'to effect all the good it is capable of;' that a similar dose of plumbago (*graphites*) will act at least from thirty-six to forty-eight days; and a like dose of phosphorus, at least forty days."

Hahnemann was accounted an ingenuous man, and is supposed to have been sincere when he proclaimed these things. He has had honest and learned followers who have adopted them in good faith, and are practising upon them every day. When we object to them, that it is impossible that doses so attenuated can operate, the appeal is continually to experience. Besides, they tell us that these wonderful virtues are imparted by the "frictions and shakings" to which they subject their medicines; and that while they cure diseases if prepared in their peculiar manner, they are inert without such preparation. This, however, does not render the matter any easier of credence. If it is impossible to credit the efficacy of the decillionth of a grain of charcoal, when pounds might be taken with impunity, except for its mechanical disturbance, it is not a whit easier to believe that poundings in an unglazed mortar, and scrapings with a spatula, and shakings in vials with so many drops of water, can add to its efficacy. Clearly, it is impossible for most minds; unhappily, not for all. Some there are that soar above all difficulties, and can believe anything.

But this is not all that homœopathy requires us to accept; nor is it the worst. All this, it is asserted, has for its basis experience and experiment; but there is an important part of the system which is essentially hypothetical, and which even the disciples of Hahnemann find it difficult to assent to.— This is his doctrine of the origin and nature of chronic diseases, of which Dr. Forbes gives the following account:

"Hahnemann maintains that all these, or with hardly an exception, are derived from three cutaneous diseases, *syphilis*, *sycosis*, and *psora*, or common itch. Of the whole class of chronic diseases, he attributes one-eighth part to the two former maladies, and the remaining seven-eighths to the last. In nearly all chronic diseases, then, the real object of consideration with the physician, and the thing to be cured, is not the ostensible diseases, but their all-pervading cause and basis, the psora, or itch. This psora he considers to be originally a disease of the whole system, which only shows itself locally on the surface in its progress. If it be cured in this form by local means, it infallibly gets worse internally, and may long subsist in this condition—for many years even—before it puts on the semblance of any formal chronic disease. In its amorphous state it may be so latent, that, although existing for years, the patient is entirely unaware of being out of health. Often, however, it is productive of a vast variety of obscure symptoms, which are seldom attended to, but which are recognizable by the adept. It is curable in this state; but it is seldom submitted to cure until it has declared itself under the guise of some more formal chronic malady. Then the patient seeks relief from medicine; and wo be unto him if he is treated by an allopath, or even by a homœopath on the general principles of homœopathy. No: it is not sufficient that the remedies prescribed are *similia similibus* simply; they must be taken from a special circumscribed class, named *anti-psoric*, discovered by Hahnemann to be gifted with a specific virtue for the cure of the itch and the itch diseases. It is laid down as an axiom, that *nature cannot possibly cure any of these chronic diseases;* but that, unless treated properly, that is, homœopathically and anti-psorically, they must infallibly get worse until they end in death. And it would seem that, although always curable by the proper remedies, they are as chronic in their cure as in their nature. The anti-psorics may work *tuto et jucunde*, but hardly *cito*, since we find Hahnemann declaring that no one but a quackish ignoramus can fancy that a disease of so long standing can be cured in a few weeks; or, if so cured, it is only for a time, to burst out with ten-fold fury by and by."

Now the whole of this is so purely fanciful, so monstrously absurd, that one has an impression as he reads it, that Hahnemann, when he put forth such nonsense, must have been experimenting upon the credulity of our race. If we should come for the first time upon such stuff in a book which had still to pass its ordeal before the public, and should fail to remember what follies and absurdities men have believed in, we would be amused at the hardihood of its author, and should not hesitate to predict, that it would be rejected by the whole world with loathing and contempt. And yet even this *quinta pars* of German foolery has found believers, or men who profess to believe it, in all the walks of society, and in our own profession. Such persons scorn the dictates of reason, and it is a waste of time to expend argument upon them. Resting securely upon the teachings of a false experience, they have a single answer to all our objections; but that chances to be a very specious one—*that they cure diseases by their system.* Demonstrate that charcoal, silica, and oyster-shells are among the most inert of substances—render their practice as ridiculous as you please, they have the standing answer—"vain declamations must cease in presence of an experience that cannot err; our patients recover, taking oyster-shells and charcoal."

We say this answer is specious; it is one which the multitude will always deem satisfactory; yet long ago it was announced by the Father of medicine, "*experientia fallax*," and most signally does the observation of every day confirm the truth of the apothegm. The Steam-doctor, the Indian-doctor, the Faith-doctor, the Mesmerizer, all appeal to "an experience that cannot err;" and amulets, charms, and the royal touch, claimed to rest upon the same impregnable basis. How long before men will be taught the truth of the saying, "*post hoc, non ergo propter hoc?*" The homœopathists point triumphantly to the results of their practice. It cannot be otherwise than profitable to see how the account between them and the regular practitioners stands. Dr. Forbes makes the following exhibit of it, which the former party is very sure not to complain of:

"The Hospital of the Sisters of Charity at Vienna was opened in 1832. It is situated in a healthy suburb, and has thus advantages over the great general hospital of the same city. It contains at present upwards of fifty beds. In the beginning of 1835, the management of the hospital was committed to Dr. Fleischmann, and since that period all the patients have been treated according to the homœopathic system exclusively. In the Introduction to the Study of Homœopathy, by Drs. Drysdale and Russel, there is a translation of a report of Dr. Fleischmann, exhibiting a tabular view of the cases treated at this hospital during eight years—from the beginning of 1835 to the end of 1843. The total number of patients treated was 6551, and the following are the general results:

| | |
|---|---|
| Remaining from 1834 - - - - | 27 |
| Admitted - - - - - - | 6524 |
| Cured - - - - - - - | 5980 |
| Dismissed uncured - - - - - | 112 |
| Died - - - - - - - | 407 |
| Remaining - - - - - - | 50 |

"The list includes all the usual diseases, acute and chronic, found in hospitals, and some surgical cases. The following extract shows the number and events of some of the most important and best marked diseases:

| | Admitted | Cured. | Uncured. | Died. | Remov. |
|---|---|---|---|---|---|
| Abscess of the brain | 3 | .. | .. | 3 | .. |
| Apoplexy | 9 | 4 | 2 | 3 | .. |
| Cancer of stomach and uterus | 5 | .. | 2 | 3 | .. |
| Amenorrhœa and Chlorosis | 90 | 89 | .. | .. | 1 |
| Ascites | 14 | 10 | 1 | 3 | .. |
| Diarrhœa | 114 | 112 | .. | 2 | .. |
| Dysentery | 44 | 42 | .. | 2 | .. |
| Erysipelas of the face | 181 | 177 | 1 | 2 | 1 |
| Fever, excluding typhus | 1036 | 1007 | 1 | 17 | 11 |
| Typhus, abdominalis | 819 | 669 | 2 | 140 | 8 |
| Influenza | 52 | 51 | .. | 1 | .. |
| Dyspeptic affections | 173 | 172 | .. | .. | 1 |
| Gout, acute and chronic | 102 | 97 | 1 | 4 | .. |
| Headaches, various | 61 | 61 | .. | .. | .. |
| Articular inflammations | 211 | 203 | .. | 2 | 6 |
| Meningitis | 17 | 15 | 1 | 1 | .. |
| Bronchitis | 15 | 15 | .. | .. | .. |
| Ophthalmia | 51 | 30 | 1 | .. | .. |
| Endocarditis | 29 | 29 | .. | .. | .. |
| Pericarditis | 2 | 2 | .. | .. | .. |
| Enteritis | 6 | 1 | .. | 5 | .. |
| Pneumonia, | 300 | 280 | .. | 19 | 1 |
| Peritonitis | 105 | 100 | .. | 5 | .. |
| Pleuritis | 224 | 221 | .. | 3 | .. |
| Measles | 25 | 23 | .. | 2 | .. |
| Phthisis | 98 | .. | 27 | 71 | .. |
| Rheumatism, acute and chronic | 188 | 188 | .. | .. | .. |
| Scarlatina | 35 | 31 | .. | 2 | 2 |
| Small-pox | 136 | 120 | .. | 11 | 5 |
| Tonsillitis | 300 | 299 | .. | .. | 1 |

"It is well known to all physicians accustomed to statistical inquiries, that, without a minute classification of the individual diseases included in any general report of cases, showing the sex, age, condition of patients, the precise character or *genius* of the prevailing diseases, the season, the date of the disease when brought under treatment, &c., &c., no trustworthy comparison can be instituted between any two lists of diseases, however similar in name, and although occurring in the same locality. The difficulty of comparison, will, of course, be considerably enhanced, when the countries, nature of the localities, general habits, &c., &c., of the patients, are different in the two cases. It would, therefore, lead to no useful purpose to institute any close comparison of Dr. Fleischmann's bare skeleton tables with any similar tables of diseases treated allopathically in this country or elsewhere. The conclusions deducible from such a comparison, whether for or against either mode of treatment, could not be admitted as of any positive weight in settling the practical question at issue. To enable us to do this effectually, we would require from each party an incomparably greater number of cases, observed and treated through a long period of time, and each disease discriminated, and all classified according to the rigid requirements of statistics. We do not, however, mean to say that such lists as those of Dr Fleischmann's are unworthy of notice and incapable of furnishing any information of consequence. This is not the case. Although yielding us no positive results of such data as science demands, they unquestionably furnish us with isolated facts of great value, and even supply materials which may be worked into such rude approximations to truth, as medicine has, alas, been too long content withal. These tables, for instance, substantiate this momentous fact, that all our ordinary curable diseases are cured, in a fair proportion, under the homœopathic method of treatment. Not merely do we see thus cured all the slighter diseases, whether acute or chronic, which most men of experience know to be readily susceptible of cure under every variety of treatment and under no treatment at all; but even all the severer and more dangerous diseases, which most physicians, of whatever school, have been accustomed to consider as not only needing the interposition of art to assist nature in bringing them to a favorable and speedy termination, but demanding the employment of prompt and strong measures to prevent a fatal issue in a considerable proportion of cases. And such is the nature of the premises, that there can hardly be any mistake as to the justness of the interence. Doctor Fleischmann is a regular, well-educated physician, as capable of forming a true diagnosis as other practitioners, and he is considered by those who know him as a man of honor and respectability, and incapable of attesting a falsehood. We cannot, therefore, refuse to admit the accuracy of his statements as to matters of fact; or, at least, to admit them, with that liberal subtraction, from the favorable side of the equation, which is required in the case of all statements made by the disciples and advocates of new doctrines. Even after this rectification, we see that enough remains to justify the inference above deduced. No candid physician, looking at the original report, or at the small part of it which we have extracted, will hesitate to acknowledge that the resuls there set forth would have been considered by him as satisfactory, if they had occurred in his own practice. The amount of deaths in the fevers and eruptive diseases is certainly below the ordinary proportion; but, for reasons already stated, no conclusions favorable to homœopathy can be thence deduced. It seems, however, reasonable to infer that, even in these cases, the new practice was not less favorable to the cure than the ordinary practice. In all such cases, experienced physicians have been long aware that the results, as to the mortality, are nearly the same under all varieties of allopathic treatment. It would not surprise them, therefore, that a treatment like that of homœopathy, which they may regard as perfectly negative, should be fully as successful as their own. But the results presented to us in the severer internal inflammations, are certainly not such as most practical physicians would have expected to be obtained under the exclusive administration of a thousandth, a millonth, or a billionth part of a grain of phosphorus, every two, three or four hours. It would be very unreasonable to believe that, out of 300 cases of pneumonia, 224 cases of pleurisy, and 105 cases of peritonitis, (in all 629 cases,) spread over a period of eight years, *all* the cases, except the fatal ones, (27 in number), were slight, and such as would have seemed to us hardly requiring treatment of any kind. In fact, according to all experience, such could not be the case. But, independently of this *a priori* argument, we have sufficient evidence to prove that many of the cases of pneumonia, at least, were severe cases. A few of these cases are reported in detail by Dr. Fleischmann himself, and we have ourselves had the statement corroborated by the private testimony of a physician (not a homœopath) who attended Dr. F.'s wards for three months. This gentleman watched the course of several cases of pneumonia and traced their progress by the physical signs, through the different stages of congestion, hepatization, and resolution, up to a perfect cure, within a period of time which would have appeared short under the most energetic treatment of allopathy.

"In examining Dr. Fleischmann's report, the sagacious physician will not fail to be struck by the fact, that the relative proportion of cures, and the relative mortality of the different diseases, one to another, are precisely the same as he is accustomed to see in his own practice. Slight diseases are all cured by the homœopathist, as by the allopathist; dangerous maladies kill a considerable proportion of the patients of both; very dangerous ones, a still larger proportion; and the class of diseases which all true observers and honest reporters have declared rebellious to their most strenuous medical efforts, are found to occupy the same black column in the tables of the old and new school.

"Thus: the cases of dyspepsia (173), the cases of headache (61), the cases of chlorosis (90), the cases of tonsillitis (300), the cases of simple rheumatism (188), the cases of bronchitis (15), are ALL *cured;* while we have 1 death in 52 cases of influenza, 2 deaths in 114 cases of diarrhœa, 2 deaths in 181 cases of erysipelas of the face, 2 deaths in 211 cases of arthritis. As we advance to the still more dangerous class of cases, we find the loss proportionably greater: thus, out of 44 cases of dysentery we have 2 deaths, out of 9 cases of apoplexy we have 3 deaths; out of 14 cases of ascites 3 deaths, and 1 not cured; out 1036 cases of ordinary fevers we have 17 deaths, while out of 819 cases of typhus we have 140 deaths; out of 524 cases of pneumonia and pleurisy we have 22 deaths; out of 105 cases of peritonitis 5 deaths, out of 136 cases of small-pox 11 deaths, out of 35 cases of scarlatina 2 deaths, and out of 6 cases of enteritis 5 deaths; while *all* the cases of cancer (5), *all* the cases of abscess of the brain (3), and *all* the cases of phthisis (98), are either registered as fatal, or as 'dismissed uncured'—which, of course, means the same thing. The only cases in the list which do not seem, at first sight, to come within the above category, are the cases of endocarditis (31), which are all reported as cured. These are, no doubt, severe diseases; and this may seem an uncommon amount of success; yet, when it is considered that the number of cases is not great, that the diagnosis of endocarditis, and even of pericarditis, is less easy and certain than that of many other diseases, and that it is not so much in their primary condition as in their ultimate effects, that these diseases are dangerous, we believe that even the degree of success here recorded cannot, in fairness be admitted as any deviation from the ordinary course of events in allopathic medication.

"The remarks above made are even of more importance, in relation to the general subject now under consideration, than they may seem to be at first. They not only show that the *kind* of successes and failures experienced by the homœopathists, is precisely the same as that experienced by the allopathists; but they also seem to show that the medication of the former can boast of no *peculiar* virtue whereby it can achieve triumphs in fields altogether forbidden by the latter. Under the influence of medicines, all of which must be considered *new*—new absolutely, or new in their form, mode of administration, and principle of action—we would have hardly expected to find the old relations of curability and incurability exactly preserved. Does not this fact, common to both, seem to point to a *community of power, or want of power*, in the two classes of agents, rather than to a specialty of action and potency in one?"

This is a long extract, and the last that we shall make from the work. It contains much matter for grave consideration. We gather from it, and from other passages in the volume, that the author does not cherish the most exalted notions of the efficacy of medicine. It is painful to perceive this growing distrust in the minds of physicians as they advance in age,—and we believe the change is not uncommon in the experience of practitioners. From being too sanguine of the potency of remedies, at the opening of their career, they come, in the end, to be too skeptical of their efficiency. It cannot be questioned that Dr. Forbes is verging towards this latter extreme, and hence it does not surprise us to learn that some of his neighbors are hinting, that it begins to be time that he had abandoned the practice of physic, in company with that old Edinburgh friend of his whom he quotes with approbation, as saying, that "for two years he had cured his patients with less than the homœopathists, viz., with *nothing at all!*" Cicero wondered how two sooth-sayers could look one another in the face without laughing. We should suppose two old fashioned practitioners of medicine, agreeing in such an estimate of the powers of their art, could hardly meet without losing their accustomed gravity of face.

But let us be just to Dr. Forbes. If his confidence in regular medicine is not great, he has no faith at all in homœopathy, which, he says, "has often failed to cure in cases in which allopathy did effect cures." He considers that the system is "calculated to destroy all scientific progress in medicine, and to degrade the minds of those who practise it,"—engaged exclusively as they are in search for and adaptation of specific remedies to symptoms; that its direct tendency is to sever medicine from the sciences, and establish it as a mere art, "thus converting physicians from philosophers to artisans." And with this conception of its character and tendencies, he conceives it to be the duty of "all who regard the prosperity and dignity of true art, to resist its progress." Has he thrown any obstacle in the line of its march by penning the article under review? We fear not. We greatly apprehend, on the contrary, that he has rather aided its progress.

If such then is his estimate of homœopathy, what is his explanation of the fact, that so many diseases are cured under its ministration? The answer is evident—*Nature effects the cures;* homœopathy does just nothing, and in an open field the *vis medicatrix* triumphs; making good the words of the poet—

> "For Nature then has room to work her way;
> And doing nothing often has prevailed,
> When ten physicians have prescribed and failed."

In this aspect, Dr. Forbes believes homœopathy has contributed something to scientific medicine; it has instituted a grand natural experiment in therapeutics which, as it is "likely to be prolonged through an indefinite period," will supply us, at last, with "a true *natural history of human diseases*, and the means of ascertaining the actual powers of nature in relieving or removing them." There is some truth in this view of the subject, and it is precisely at this point that the claims of the "system" upon the respect of medical men, terminate. Its merits "have this extent—no more." It has taught us how powerful is nature in the cure of diseases when her efforts are seconded by a proper regimen; and in estimating the resources of homœopathy we must never overlook the dietetic principles upon which its practitioners wisely insist as a cardinal point of their therapeutics. In the stringency of their system we never read of anything that came up to them, except the "diet absolute" of the physician to the Governor of Barataria, in Don Quixotte, the famous Doctor Pedro Rezio de Aguera. The hostility of this worthy disciple of Hippocrates to fruit, "as being too moist," and to "meat as being too hot;" to roasted partridges because his great master condemned them, and to "stewed coneys, because they are a sharp-haired food," did not exceed the enmity of the homœopathists to many of the common articles of diet. Thus, in his Organon, we find Hahnemann proscribing the following things:

"Coffee, tea, beer, drinks containing aromatics, spiced chocolate, scents and perfumeries of all sorts, tooth-powders (liquid or pulverized) containing aught medicinal, perfumed bags, high-seasoned meats, ices and pastry flavored with aromatics, all herbs and roots having medicinal properties, cheese, meats too long kept, pork, goose, duck, too young veal. The following things are also prohibited: over-indulgence at table of all kinds, too much salt or sugar, all spirituous drinks, over-heated rooms, sedentary life, passive exercise on horseback or in a carriage, sleep after dinner, sexual pleasures, exciting books, uncleanliness, anger, vexation, scorn, exciting play, over-exertion of mind, marshy districts, confined localities where the air is stagnant, &c."

We say, the homœopathists wisely insist upon their rules of diet, and no one we suppose will dispute the truth of the remark. The following case, illustrative of it, occurred to us during the last summer, and the experience of every physician who has been long in practice, no doubt, will furnish him with similar instances. A man, about 30 years of age, had removed a short time before from the country, where he had been treated actively with mercurial purgatives for a disease of the liver. His complexion was pale, and, altogether, he looked to us like one who had taken too much medicine. Accordingly we advised him to abandon drugs, except so far as was requisite to keep the bowels open, and to rely upon diet for the cure of his complaint. We saw him occasionally, and the system was evidently working well. This spring we met him, after several months, and he had grown fat, his complexion having become good, and his countenance wearing the peculiar expression of health. We were too curious to let him pass without endeavoring to learn how so happy a change had been wrought in his condition. His reply was very brief, but very satisfactory—"*I did'nt get well, sir, till I quit all medicines, and coffee and tobacco.*" What a case this would have been for the homœopathists, and how they would have exulted in its termination, as showing the superiority of "sepia, charcoal, and oyster-shells," over rhubarb, calomel and aloes! It is an instructive case, and certainly teaches that medicine is not always good.

But so saying, do we not condemn ourselves also? If we set down homœopathy, with Dr. Forbes, as a *natural history of diseases*—as showing what they are when left to themselves, the corollary is one not flattering to our professional vanity. That the sick often recover under the treatment of these practitioners it is useless to deny; that many acute and dangerous diseases have a favorable termination when subjected to no system of therapeutics but theirs, must be admitted; that they are *cured* homœopathically—by the millionth of a grain of nux vomica, or the quadrillionth (1,000000,-000000,000000,000000,000000,000000) of a grain of belladonna, or the decillionth of a grain of *sepia*—we certainly cannot admit. What then is the inference? Clearly, none other than this—that the curative powers of nature, unaided by art, would often accomplish what we now regard as triumphs of medical skill. It is one which we feel no pleasure or pride in adopting; but if it is true, we do not profit at all by shutting our eyes to the truth. Medicine is not perfect; but there is enough about it intrinsically valuable and excellent to bear the touch of the most rigid inquiry. In adhering to it, after admitting all its deficiencies, we feel assured that, in the language of Dr. Forbes, we "are embracing a system which contains a considerable amount of truth, and a yet greater amount of good; and which, above all, is, or may be made, in its exercise, consonant with the principles of science, and is capable of indefinite improvement; while in rejecting homœopathy, we consider that we are discarding what is at once bad—useless to the sufferer and degrading to the physician."

A great *Reformation* in practical medicine, Dr. Forbes believes to be impending. This is his "*Young Physic*," who, in his estimation, has a task only a little less difficult than that of the infant Hercules to perform. We have not time to follow him through the Augean Stable he describes, and repeat his account of the nuisances in it to be abated.

With a good deal of truth, there is a large admixture of what is not true in this vigorous little volume on this subject, as well as some others—much that appears to be candid and fair, but much also that is exaggerated—a good deal of tenderness towards homœopathy, and not a little harshness towards scientific medicine; and it may be questioned, upon the whole, whether the author, by the representation he has given of the two systems, has conferred any real benefit upon his profession.

